# Comparison of the Effects of Denosumab and Alendronate on Cardiovascular and Renal Outcomes in Osteoporotic Patients

**DOI:** 10.3390/jcm8070932

**Published:** 2019-06-28

**Authors:** Tsuen-Wei Hsu, Chien-Ning Hsu, Shih-Wei Wang, Chiang-Chi Huang, Lung-Chih Li

**Affiliations:** 1 Division of Nephrology, Department of Internal Medicine, Kaohsiung Chang Gung Memorial Hospital and Chang Gung University College of Medicine, Kaohsiung 833, Taiwan; 2 Department of Pharmacy, Kaohsiung Chang Gung Memorial Hospital and Chang Gung University College of Medicine, Kaohsiung 833, Taiwan; 3 School of Pharmacy, Kaohsiung Medical University, Kaohsiung 807, Taiwan; 4 Institute for Translational Research in Biomedicine, Kaohsiung Chang Gung Memorial Hospital and Chang Gung University College of Medicine, Kaohsiung 833, Taiwan

**Keywords:** alendronate, cardiovascular disease (CVD), chronic kidney disease (CKD), denosumab, osteoporosis

## Abstract

A correlation between impaired bone metabolism, chronic kidney disease, and cardiovascular diseases (CVD) has been suggested. This study aimed to compare the effects of denosumab and alendronate, two anti-resorptive agents, on cardiovascular and renal outcomes in osteoporotic patients. Propensity score-matched cohort study comparing denosumab to alendronate users between January 2005 and December 2017 was conducted from a large medical organization in Taiwan. Risks of CVD development and renal function decline were estimated using Cox proportional hazard regression. A total 2523 patients were recruited in each group. No significant difference in cardiovascular events was found between the two groups over a 5-year study period. Stratified analysis results showed that denosumab was likely to exert protective effects against composite CVD in patients with medication possession rate ≥60% (adjusted hazard ratio (AHR), 0.74; *p* = 0.0493) and myocardial infraction (AHR, 0.42; *p* = 0.0415). Denosumab was associated with increased risk of renal function decline in male patients (AHR, 1.78; *p* = 0.0132), patients with renal insufficiency (AHR, 1.5; *p* = 0.0132), and patients with acute kidney injury during the study period (AHR, 1.53; *p* = 0.0154). Conclusively, denosumab may exert cardiovascular benefits in patients with good adherence but may have renal disadvantages in certain conditions and thus must be used with caution.

## 1. Introduction

Osteoporosis and cardiovascular diseases (CVD) are the major causes of mortality and morbidity in the elderly [1]. Evidence has shown that both osteoporosis and CVD share similar pathophysiology and risk factors; thus, patients with osteoporosis are at higher risk for development of major CVD, such as ischemic heart disease and stroke, compared with the general population [1,2]. A link between fracture, low bone mineral density (BMD), and vascular calcification has been proposed in patients with osteoporosis, and all were associated with increased risk of cardiovascular mortality [3,4]. Furthermore, moderate to severe renal insufficiency was associated with increased risk of fracture and decreased BMD [5,6].

Renal insufficiency is independently associated with increased risk of cardiovascular events. Patients with chronic kidney disease (CKD) are at 2 to 16 times higher risk of CVD, compared with patients with normal estimated glomerular filtration rate (eGFR) [7]. Vascular calcification has been proven as a characteristic change in the vessels of CKD, and is associated with increased risk of cardiovascular mortality and CKD progression [8]. The prevalence of vascular calcification in CKD patients has been reported to be 47% to 92% [9]. Controlling abnormalities in serum levels of phosphorus, calcium, vitamin D, and parathyroid hormone has been recommended to prevent the progression of vascular calcification and cardiovascular morbidity and mortality in patients with CKD [10].

The mechanism of vascular calcification is complex, and it involves the interaction of various signaling pathways [11]. Recent studies revealed that osteoporosis and vascular calcification may share common pathogenetic mechanisms involving bone morphogenetic proteins and the osteoprotegerin (OPG)/receptor activator of nuclear factor-ĸB (RANK)/RANK Ligand (RANKL) pathway [3]. RANKL is a member of the tumor necrosis factor superfamily [12], which plays a key role in bone metabolism by stimulating osteoclast formation, activation, and survival [13,14]. Research has shown that RANKL also plays an important role in the progression of atherosclerosis and vascular calcification [15]. Low level of serum RANKL is also associated with an increased risk of CVD in older women and men [16].

Denosumab is a fully human monoclonal antibody that acts as an OPG mimicker directed against the receptor activator of RANKL. By blocking the binding of RANKL to RANK, denosumab decreases the number and activity of osteoclasts, decreases bone resorption, and increases BMD [14]. In an animal study, denosumab inhibits aortic calcium deposition and prevents bone loss following glucocorticoid exposure in human RANKL knock-in mice [17]. In the Fracture Reduction Evaluation of Denosumab in Osteoporosis Every 6 Months (FREEDOM) trial, a randomized double-blinded trial, denosumab improves BMD and reduces fracture risk in elderly patients (60–90 years old) with an eGFR ≥ 15 mL/min/1.73 m^2^ [18]. For all these reasons, we hypothesized that denosumab may play a role in reducing cardiovascular events and renal function progression through protection against BMD loss and slow progression of vascular calcification [19,20].

Although evidence shows that denosumab prevents fractures in patients with osteoporosis, limited information is available regarding its effects on CVD risks and renal function progression. The present study aimed to assess the effects of denosumab on CVD prevention and renal function in osteoporotic patients, compared to those of alendronate, a bisphosphonate agent and the first-line treatment for osteoporosis. As alendronate acts as an anti-resorptive agent via different mechanism from denosumab [21,22] and had been used in many large randomize control trials [19,20], it is rational to compare denosumab to alendronate.

## 2. Materials and Methods

### 2.1. Study Design and Study Cohort

This was a retrospective cohort study using the Chang Gung Research Database (CGRD), which is an electronic health record dataset derived from a group of Chang Gung Memorial Hospitals (CGMH) in Taiwan. CGMH is the largest medical center in Taiwan that provides approximately 10%–12% of the healthcare services of the Taiwan national health insurance (NHI) program [23]. The Taiwan NHI program is a compulsory, single-payer health insurance program that covers over 99% of Taiwan’s entire population [24]. The CGRD contains detailed diagnosis, prescription, and laboratory test results from emergency department in both in- and outpatient settings. The study was approved by the Institutional Review Boards of the Chang Gung Medical Foundation at Taoyun, Taiwan (permit number: 201800313B0).

**Figure 1:**
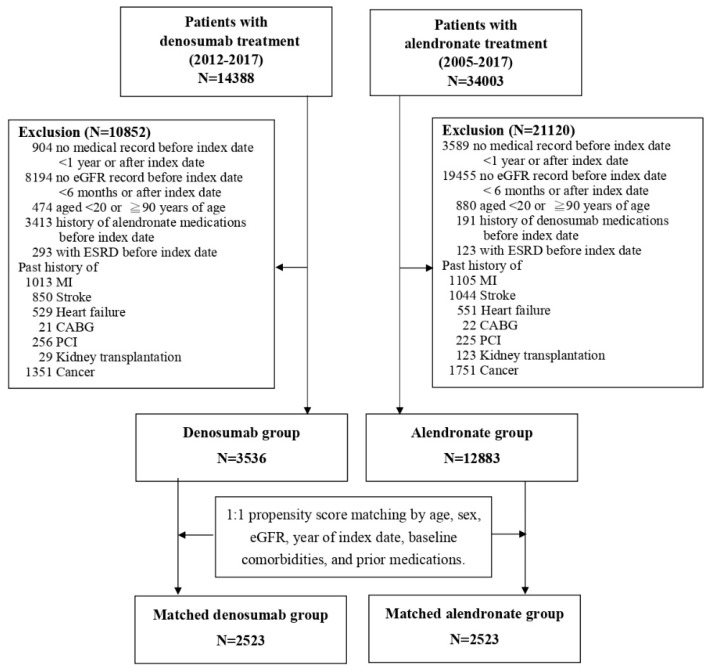
Study flowchart. Propensity score was calculated based on baseline comorbidities: Peripheral vascular diseases, dementia, pulmonary disease, connective tissue disorder, peptic ulcer, liver diseases, diabetes, diabetes complications, paraplegia, renal disease, severe liver diseases, metastatic cancer, hypertension, hyperlipidemia, abnormal thyroid function, obstructive sleep apnea and fracture, and prior medication uses (oral anticoagulants, antiplatelets, aspirin, statins, fibrates, other lipid-lowering agents, antidiabetics, angiotensin converting enzyme inhibitors (ACEI)/angiotensin receptor blockers(ARBs)/Aliskiren, diuretics, bisphosphonates, raloxifene, forteo, and calcitonin preparations). CABG: Coronary artery bypass surgery; ESRD: End stage renal disease; eGFR: Estimated glomerular filtration rate; MI: Myocardial infarction; PCI: Percutaneous coronary intervention.

Patients aged 20–89 years at the time of initiation of denosumab or alendronate between 1 January 2005 and 31 December 2017 were included in the study, and only patients having ≥1-year admission records before treatment initiation were included in the study. To assess cardiovascular health and renal outcomes, patients were excluded for lack of baseline serum creatinine (SCr) value or having preexisting medical history of chronic dialysis, kidney transplantation, myocardial infarction, congestive heart failure, ischemic stroke, and cancer. Patients receiving procedures for cardiovascular diseases, including coronary artery bypass surgery (CABG) and percutaneous coronary intervention (PCI), were also identified before treatment initiation and excluded from analyses. Further, patients without any admission records or SCr (or eGFR) values in their follow-up were excluded (Figure 1). Operational definitions and codes for disease conditions and procedures are available in Table S1.

### 2.2. Comparison Groups

Patients were classified as the denosumab or alendronate group based on the initial prescription of study medications in out- or inpatient settings. New users of denosumab were identified as patients who never had alendronate within 365 days at baseline period, and the earliest date of denosumab prescribed was defined as the index date. Same criteria were applied for new users of alendronate without prior denosumab treatment.

To minimize potential selection bias, 1:1 propensity score matching (PSM) without replacement was employed to establish matched comparison groups. To adjust for time-varying treatment exposure, that is, patients in the denosumab group switched to alendronate or vice versa during the follow-up period, outcome event was censored upon treatment switch.

### 2.3. Outcomes

The incidence of the composite of major CVD, including myocardial infarction, congestive heart failure, and ischemic stroke, was assessed based on hospital discharge diagnoses. Individual outcomes of composite CVD were also analyzed. Renal outcomes were assessed by the mean changes in eGFR from baseline and by incident of eGFR decline ≥30% of baseline. eGFR was assessed every 6 months using the Modification of Diet in Renal Disease (MDRD) equation (175 × SCr − 1.154 × age − 0.203 × 0.742 (if female), mL/min/1.73 m^2^) [25]; operational definition of individual study outcome is available as a Supplementary Material (Table S1).

### 2.4. Study Covariates

Baseline variables considered in the analyses included patient’s demographics; comorbid conditions, including items in the Charlson comorbidity index (CCI) and cardiovascular disease risk associated with preexisting hyperlipidemia; procedures of CABG and PCI; and prior medication use: Anti-thrombotic agents (anti-coagulants, anti-platelets), lipid-lowering agents, glucose-lowering agents, antihypertensive agents and other osteoporosis therapy other than study drugs. Prior use of medication with ≥28 days of supply was identified ≤365 days before the index date. Concomitant use of these medications with ≥28 days of supply was identified between the index date and the earliest date of event of interest.

Because the occurrence of acute kidney injury (AKI) is associated with deterioration of renal function, AKI defined by the Kidney Disease Improving Global Outcomes (KDIGO) criteria was assessed during follow-up period [26]. Operational definitions of disease conditions and medications uses are available as Supplementary Material (Table S1).

Medication adherence of denosumab (60 mg/mL/syringe, one unit per 6 months) was classified by the number of syringes in the follow-up, namely as <60% vs. ≥60% medication possession rate (MPR) in every half-year period (e.g., 3 doses/2 years (=expected 4 doses) = 75%). The MPR of alendronate (alendronate 70 mg/tab, alendronate-cholecalciferol 70 mg–70 mcg/tab, one unit per week) in the follow-up was calculated in a 7-day interval over the follow-up period.

### 2.5. Statistical Analysis

Descriptive statistical analysis results of continuous variables were reported as mean and standard deviation (SD) or median and interquartile range (IQR: 25th, 75th percentile), and data were summarized as n (%) for categorical variables in the study cohort. Comparisons between groups were performed using the Pearson Chi-square test for categorical variables and the Student t-tests for continuous variables.

Propensity score was calculated using logistic regression to model the probability of receipt of denosumab (or alendronate) as a function of patient characteristics in the baseline period, including sex, age at index date, year of treatment initiation, individual CCI conditions, and hyperlipidemia [27]. Standardized mean difference was used to measure covariate balance, and a value of >0.1 indicated meaningful imbalance after PSM [28].

All patients were followed up from the index date until outcome of interest, death, loss to follow up, or the latest date in the dataset (31 December 2017), whichever came first. Among patients that switched therapy, active treatment was censored at the time of switching. Time to CVD endpoint was analyzed using Kaplan–Meier with log-rank tests. Cox proportional hazard regression was employed for composite incident cardiovascular events and individual cardiovascular event.

Stratified analyses by MPR ≥60% (vs. <60%) and baseline eGFR ≥60 (vs. <60) mL/min/1.73 m^2^ were performed to assess the heterogenous effects of denosumab and alendronate. These factors were known to be associated with cardiovascular morbidity and renal outcomes. A two-sided *p*-value of <0.05 was considered statistically significant. All statistical analyses were performed using SAS 9.4 (SAS Institute, Cary, NC, USA).

## 3. Results

### 3.1. Patient Characteristics

A total of 48,391 adult osteoporotic patients receiving alendronate or denosumab were identified, 16,419 of them met the inclusion criteria (denosumab: 3536 patients, alendronate: 12,883 patients) (Figure 1). The mean age of all patients was 69.6 years, and 79.4% of the patients were female. Compared with patients initiated with alendronate, those initiated with denosumab had slightly lower eGFR (77.01 ± 26.47 vs. 72.13 ± 30.57 mL/min/1.73 m^2^) and had more history of dementia, peptic ulcer, diabetes, hypertension, hyperlipidemia, and renal disease (Table 1).

In the 1:1 PSM cohort, 5046 denosumab and alendronate-matched pairs were analyzed over a 5-year follow-up period. The baseline characteristics were well balanced in the matched groups and are presented in Table 1. There were 2128 (84.34%) patients who adhered with alendronate and 2462 (97.58%) who adhered with denosumab. During the follow-up period, most patients were concomitantly administered medications for hypertension, followed by medications for hyperlipidemia, thrombotic prevention, and diabetes; the frequency of these medications was similar between the denosumab and alendronate groups (Table 1). On the contrary, more patients in the alendronate group were concomitantly treated with other osteoporosis therapy than in the denosumab group (9.79% vs. 5.31%, respectively; *p* < 0.0001) (Table S2).

### 3.2. CVD Incidence

**Figure 2:**
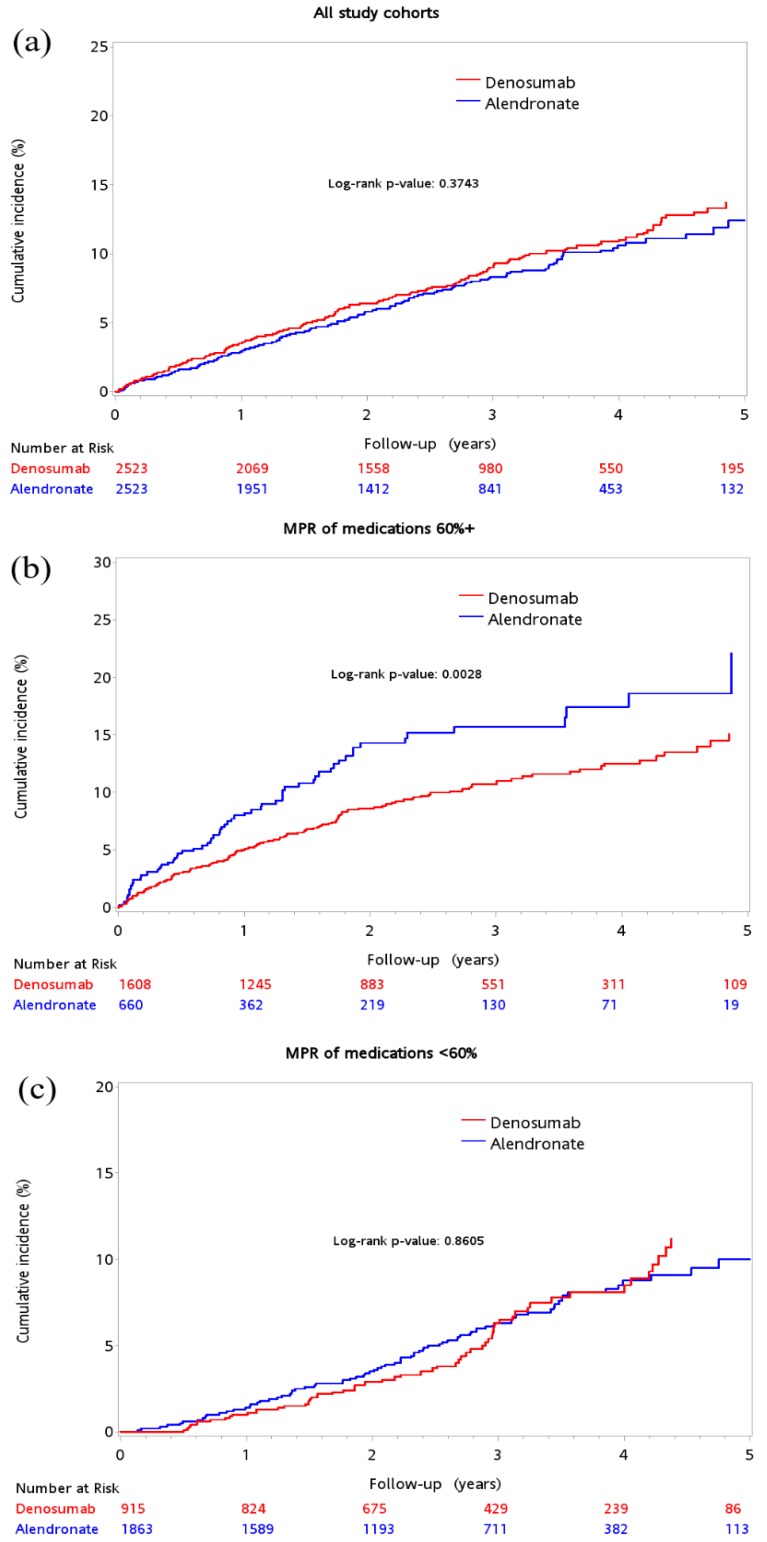
Cumulative incidence of composite cardiovascular outcomes between the denosumab and alendronate groups: All patients (**a**); patients with MPR ≥60% (**b**); patients with MPR <60% (**c**); MPR: medication possession rate.

During the 5-year follow-up period, the incident of CVD was 7.97% (*n* = 201) and 6.74% (*n* = 170) in the denosumab and alendronate groups, respectively. The event with the highest rate was ischemic stroke (4.84%), followed by congestive heart failure (2.58%) and myocardial infarction (1.01%). Table 2 lists the incidence of composite and individual CVD events in all patients, stratified by MPR. Cumulative incidence of composite CVD was similar between patients in the denosumab and alendronate groups after the 5-year follow-up (Figure 2a; log-rank test, *p* = 0.3743). However, among patients with MPR ≥60%, disease incidence was significantly lower in the denosumab group than in the alendronate group (9.08% vs. 10.3%, respectively) (Figure 2b; log-rank test, *p* = 0.0028). In patients with MPR <60%, denosumab and alendronate (6.01% vs. 5.48%) showed no difference in CVD incidence (Figure 2c); log-rank test, *p* = 0.8605).

Regarding adjustment for baseline characteristics, use of concomitant medications, and occurrence of AKI, there was a trend toward significant difference in patients with MPR ≥60%. Patients treated with denosumab had a lower risk of developing CVD than those treated with alendronate (AHR 0.74; 95% CI 0.55 to 0.99; *p* = 0.0493) (Table 3). Among patients with MPR ≥60%, those treated with denosumab, compared with those treated with alendronate, revealed lower risk of myocardial infarction development (AHR 0.42; 95%CI, 0.18–0.97; *p* = 0.0415), with AHR of 0.72 (95%CI, 0.5–1.04; *p* = 0.0805) for ischemic stroke and 0.74 (95%CI, 0.44–1.24; *p* = 0.2534) for congestive heart failure. Among patients with MPR ≥60%, baseline comorbidities and prior medication uses were closed between groups, except for higher renal, liver diseases and connective tissue disease in the alendronate group (Table S3).

### 3.3. Renal Outcomes

Kidney function decreased in both groups during the 5-year period. Patients in the denosumab group had lower eGFR at the 5th year than those in the alendronate group (62.16 ± 31.99 vs. 73.36 ± 33.55 mL/min/1.73 m^2^, respectively) (Figure S1). The median decline in eGFR from baseline was of 0.71 mL/min/1.73 m^2^ per year (IQR, −3.41, 4.90) and 0.38 mL/min/1.73 m^2^ per year (IQR, −3.30, 4.90) in the denosumab and alendronate groups, respectively.

**Figure 3:**
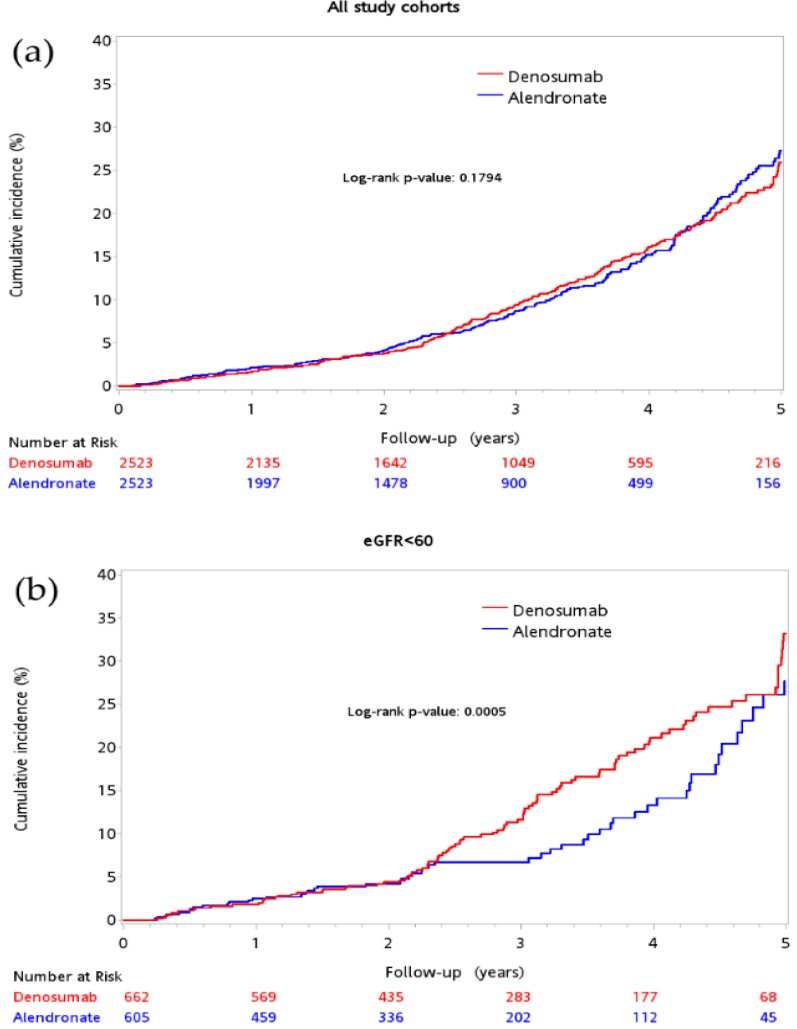

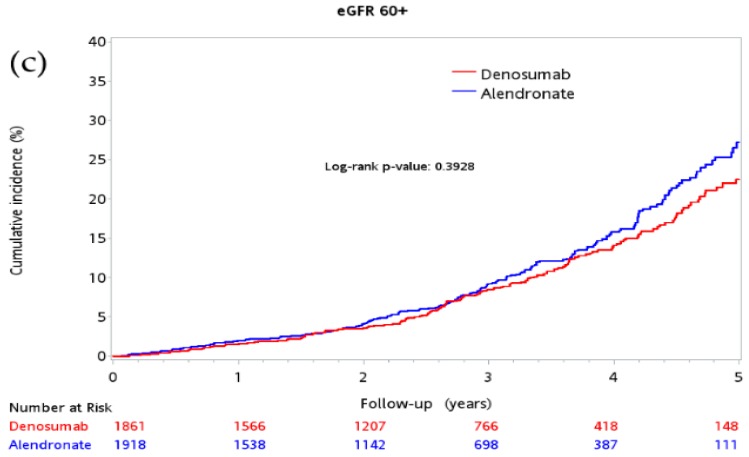
Cumulative incidence of eGFR decline ≥30% between the denosumab and alendronate groups: All patients (**a**); patients with eGFR<60 mL/min/1.73 m^2^ at baseline (**b**); patients with eGFR ≥60 mL/min/1.73 m^2^ at baseline (**c**); eGFR: estimated glomerular filtration rate.

The incidence of eGFR decline ≥30% of baseline was 10.4% in the matched cohort, 11.97% in the denosumab group, and 10.19% in the alendronate group (*p* = 0.0436) (Table 4). There was no significant difference in cumulative probability of eGFR decline ≥30% of baseline during the 5-year period between the groups (log-rank test, *p* = 0.1794) (Figure 3a). However, patients in the denosumab group showed higher cumulative event rate of eGFR decline ≥30% when baseline renal eGFR <60 mL/min/1.73 m^2^ (log-rank test, *p* = 0.0005) (Figure 3b).

**Figure 4:**
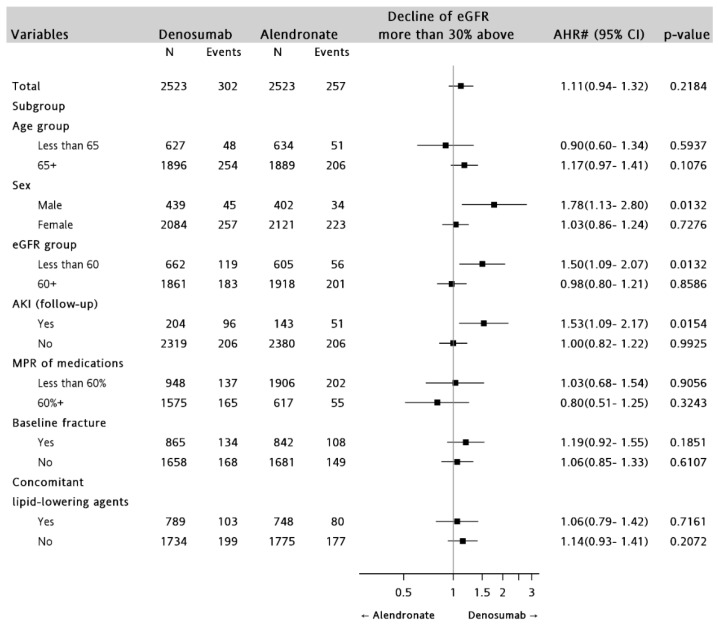
Stratified analyses of the risk of eGFR decline ≥30% between the denosumab and alendronate groups (denosumab versus alendronate). AKI: acute kidney injury; AHR: adjusted hazard ratio; eGFR: estimated glomerular filtration rate; MPR: medication possession rate. # Adjusted for age group, sex, baseline eGFR group, mean dose of drug, CVD-related comorbidities, baseline comorbidities (abnormal thyroid function, obstructive sleep apnea, and fracture), concomitant medications (anti-thrombotic agents, lipid-lowering agents, antidiabetics, antihypertensive agents, and other osteoporosis therapy).

Denosumab treatment was associated with significantly higher risk of eGFR decline ≥30% than alendronate treatment, when stratified by AKI occurrence (AHR: 1.53; 95% CI 1.09–2.17; *p* = 0.0154), male (AHR: 1.78; 95%CI 1.13–2.80; *p* = 0.0132), and baseline eGFR <60 mL/min/1.73 m^2^ (AHR 1.50; 95%CI 1.09–2.07; *p* = 0.0132); adjusted for baseline characteristics and use of concomitant medications (Figure 4).

## 4. Discussion

In the present study, denosumab and alendronate treatments revealed no difference in CVD incidence in the 5-year period. However, in patients with MPR ≥60%, denosumab treatment was associated with lower risk of myocardial infraction development. On the contrary, denosumab treatment, compared with alendronate therapy, had a trend toward poorer renal outcome in males and in patients with poor renal function at baseline and AKI episodes.

The OPG/ RANK/RANKL system has been proposed to mediate both bone remodeling and vascular calcification [29]. Although many previous studies had reported that OPG is a risk factor for progressive atherosclerosis and cardiovascular disease [30,31], the role of RANKL in the pathological mechanisms of CVD remains unclear. RANKL had been detected in thrombus materials at the site of plaque rupture in human coronary arteries [32], but not in normal vasculature [33]. Besides, lower serum RANKL level was associated with increased cardiovascular risk in patients with ischemic heart disease [34]. Although we have observed the patients for a longer period (5 years rather than 3 years), the current results and the post-hoc analyses of FREEDOM trial, which evaluated the effects of denosumab on aortic calcification progression in postmenopausal women [35], did not reveal the potential benefits of denosumab in protecting against cardiovascular morbidity in both real-world practice and trial settings.

Our study, as others’ studies [20,36], showed that patients treated with denosumab exhibited better medication adherence than patients treated with alendronate. In the stratified analysis, patients with MPR ≥60% treated with denosumab revealed protective effect against CVD development, compared with those treated with alendronate. In the FREEDOM trial, 76% finished the complete injection [18], whereas in a study by Samelson et al. [35], 2363 of patients with CVD risk score >4 and received at least one dose of denosumab were included. The protective effects of denosumab against CVD may become more significant only when the patients show higher adherence to denosumab. These findings are applicable to clinical practice. However, there is insufficient evidence to conclude that a patient adhered better to anti-resorptive therapy would has better adherence to their concomitant medications in terms of intensity or continuation during the study period. Owing to data limitation, our study did not obtain direct or indirect evidence of improved vascular calcification by X-ray or pulse wave velocity. Future studies investigating the pathophysiology of denosumab on cardiovascular events should be conducted.

There is little information regarding the effect of renal function in patients treated with denosumab, even in previous large randomized control trials of denosumab [18,19,20]. Because denosumab is not eliminated through the kidney [37,38], it is possible that scheduled renal monitoring for denosumab use was not considered in previous clinical trials. Two sequential studies of the FREEDOM trial showed no significant association between denosumab and worsening of eGFR and increased risk of CVD [35,39].

It is worth noticing that alendronate is not recommended in patients with creatinine clearance (CrCl) less than 35 mL/min [40], whereas denosumab is not restricted to patients with CKD. Recent systemic review and meta-analysis have demonstrated that denosumab increases bone mineral density and reduces fracture in renal transplant patients [41]. However, the safety of denosumab in CKD patients remains unclear [42]. In the present study, we also found that patients with renal diseases and ESRD were more in the denosumab than the alendronate group before PSM. In the matched cohort, baseline distribution of renal diseases was well balanced between treatment groups, study results showed that denosumab was associated with increased risk of worsening renal outcomes in the group of denosumab initiators with baseline eGFR <60 mL/min/1.73 m^2^. These study findings bridge the knowledge gap and will enhance the awareness of the importance of renal monitoring in denosumab users. Although proteinuria and nephrotoxic medications (e.g., analgesics) were not all included in the analysis, they are relevant to renal function deterioration and should be regularly monitored in patients (e.g., diabetes, eGFR <60, male patients) who are at risk of a rapid decline in eGFR.

Some studies showed inconsistent findings in renal outcomes between denosumab and other anti-resorptive treatments. A study reported that renal function was improved in patients with bone metastases by changing bone-modifying agent from zoledronic acid to denosumab [43]. Other randomized phase III studies in patients with multiple myeloma or bone metastasis revealed 5%–15% of adverse renal outcomes in denosumab and zoledronate treatments [44,45]; however, the definition of renal adverse events and whether the renal adverse events were attributed to chemotherapy or anti-resorptive therapy were not well explained.

Choosing proper control may be an important issue in identifying whether denosumab has renal benefits or not. It has been suggested that intravenous high-dose bisphosphonates, such as pamidronate or zoledronate, show higher renal toxicity than oral bisphosphonates [46,47]. In contrast, a review article pointed out that alendronate has no negative effect on renal function, including in patients with eGFR of as low as 15 mL/min/1.73 m^2^ [48]. Hence, it may be more reasonable to compare denosumab with alendronate. Taken together, despite its effectiveness in treating osteoporosis, denosumab should be used with caution, especially in male patients and in patients with AKI or renal insufficiency. Well-designed future studies exploring the effects denosumab on renal function in a specific population are necessary.

To our knowledge, this study is by far the largest-sized cohort study with over 5-year of follow-up for head-to-head comparison of denosumab and alendronate in patients with osteoporosis. This study allowed us to detect small differences in cardiovascular morbidity and renal outcomes between denosumab and alendronate treatments. The new user cohort design with PSM to account for confounding by indication is an important method to minimize bias. However, there was a possibility of unconsidered or unmeasured residual confounding, including smoking, alcohol, and body mass index as in many observational studies. Bone mineral density and bone turnover markers have not been available in the study setting. Moreover, because laboratory results, such as 1,25(OH)_2_ vitamin D and parathyroid hormone, are not part of routine care nor health insurance reimbursed tests in the study setting, our opportunity to evaluate the association between anti-resorptive therapy and vascular calcification and renal function was limited in this study. Lastly, the study individuals were derived from academic medical centers, and these findings might not be suitable for generalization to other settings having patients with less comorbid conditions. However, results of the current study raised intriguing questions worthy of additional investigation, such as the specific mechanisms of the cardiovascular protection of denosumab and their associations with renal function progression.

## 5. Conclusions

In this large cohort study, new denosumab users showed similar cardiovascular events as those of new alendronate users. However, in patients with adherence of ≥60% to MRP, denosumab treatment, compared with alendronate treatment, may result in significantly reduced risk of major cardiovascular events. With the popularity of denosumab use as an osteoporosis treatment in clinical practice settings, renal function status should be monitored regularly over treatment period, especially in male patients and in those with renal insufficiency at baseline.

## Figures and Tables

**Table 1:** Patient characteristics before and after matching.

	Before PSM						After PSM					
	Overall	Denosumab (*n* = 3536)		Alendronate (*n* = 12,883)		SMD	Overall	Denosumab (*n* = 2523)		Alendronate (*n* = 2523)		SMD
	*n*	*n*	(%)	*n*	(%)		*n*	*n*	(%)	*n*	(%)	
**Age at the index date, years** Mean ± SD	16,419	72.98	±9.87	68.72	±12.33	0.38	5046	71.59	±10.13	71.22	±10.23	0.04
**Sex**												
Male	3377	486	(13.74)	2891	(22.44)	0.23	841	439	(17.40)	402	(15.93)	0.04
Female	13,042	3050	(86.26)	9992	(77.56)	0.23	4205	2084	(82.60)	2121	(84.07)	0.04
**Baseline eGFR** Mean ± SD	16,419	72.13	(30.57)	77.01	(26.47)	0.17	5046	78.35	(29.65)	78.77	(26.29)	0.01
**Baseline comorbid conditions**												
Peripheral vascular diseases	237	71	(2.01)	166	(1.29)	0.06	90	47	(1.86)	43	(1.70)	0.01
Dementia	642	206	(5.83)	436	(3.38)	0.12	224	110	(4.36)	114	(4.52)	0.01
Pulmonary disease	1903	435	(12.30)	1468	(11.39)	0.03	575	292	(11.57)	283	(11.22)	0.01
Connective tissue disorder	711	167	(4.72)	544	(4.22)	0.02	260	123	(4.88)	137	(5.43)	0.03
Peptic ulcer	2917	746	(21.10)	2171	(16.85)	0.11	992	489	(19.38)	503	(19.94)	0.01
Liver diseases	1981	496	(14.03)	1485	(11.53)	0.07	710	353	(13.99)	357	(14.15)	0.00
Diabetes	3738	963	(27.23)	2775	(21.54)	0.13	1188	588	(23.31)	600	(23.78)	0.01
Diabetes complications	968	298	(8.43)	670	(5.20)	0.13	374	182	(7.21)	192	(7.61)	0.02
Paraplegia	142	20	(0.57)	122	(0.95)	0.04	40	19	(0.75)	21	(0.83)	0.01
Renal disease	1158	531	(15.02)	627	(4.87)	0.34	443	231	(9.16)	212	(8.40)	0.03
Severe liver diseases	82	24	(0.68)	58	(0.45)	0.03	25	13	(0.52)	12	(0.48)	0.01
Metastatic cancer	14	2	(0.06)	12	(0.09)	0.01	3	2	(0.08)	1	(0.04)	0.02
Hypertension	7195	1867	(52.80)	5328	(41.36)	0.23	2388	1208	(47.88)	1180	(46.77)	0.02
Hyperlipidemia	3849	1078	(30.49)	2771	(21.51)	0.21	1428	718	(28.46)	710	(28.14)	0.01
Thyroid function abnormal	280	96	(2.71)	184	(1.43)	0.09	108	53	(2.10)	55	(2.18)	0.01
Obstructive sleep apnea	131	84	(2.38)	47	(0.36)	0.17	98	55	(2.18)	43	(1.70)	0.03
Fracture	5871	1358	(38.40)	4513	(35.03)	0.07	1707	865	(34.28)	842	(33.37)	0.02
**Prior medications**												
Oral anticoagulants	247	85	(2.40)	162	(1.26)	0.09	118	59	(2.34)	59	(2.34)	0.00
Anti-platelet agents	2623	584	(16.52)	2039	(15.83)	0.02	792	404	(16.01)	388	(15.38)	0.02
Aspirin	2033	447	(12.64)	1586	(12.31)	0.01	626	318	(12.60)	308	(12.21)	0.01
Statins	2825	843	(23.84)	1982	(15.38)	0.21	1094	551	(21.84)	543	(21.52)	0.01
Fibrates	355	83	(2.35)	272	(2.11)	0.02	110	61	(2.42)	49	(1.94)	0.03
Other lipid-lowering agents	68	17	(0.48)	51	(0.40)	0.01	24	12	(0.48)	12	(0.48)	0.00
Anti-diabetic agents	3072	768	(21.72)	2304	(17.88)	0.10	1000	490	(19.42)	510	(20.21)	0.02
ACEI/ARB/Aliskiren	4293	1170	(33.09)	3123	(24.24)	0.20	1533	773	(30.64)	760	(30.12)	0.01
Diuretics	800	151	(4.27)	649	(5.04)	0.04	194	99	(3.92)	95	(3.77)	0.01
Bisphosphonates	18	4	(0.11)	14	(0.11)	0.00	5	2	(0.08)	3	(0.12)	0.01
Raloxifene	558	188	(5.32)	370	(2.87)	0.12	178	97	(3.84)	81	(3.21)	0.03
Teriparatide	186	71	(2.01)	115	(0.89)	0.09	74	36	(1.43)	38	(1.51)	0.01
Calcitonin preparations	368	22	(0.62)	346	(2.69)	0.16	40	20	(0.79)	20	(0.79)	0.00
**Year of index date**												
2012	1463	324	(9.16)	1139	(8.84)	0.01	608	314	(12.45)	294	(11.65)	0.02
2013	1446	628	(17.76)	818	(6.35)	0.36	1065	526	(20.85)	539	(21.36)	0.01
2014	1342	686	(19.40)	656	(5.09)	0.45	1008	493	(19.54)	515	(20.41)	0.02
2015	1458	786	(22.23)	672	(5.22)	0.51	1126	567	(22.47)	559	(22.16)	0.01
2016	1077	634	(17.93)	443	(3.44)	0.48	789	397	(15.74)	392	(15.54)	0.01
2017	710	478	(13.52)	232	(1.80)	0.45	450	226	(8.96)	224	(8.88)	0.00

ACEI: angiotensin converting enzyme inhibitors; ARBs: angiotensin receptor blockers; eGFR: estimated glomerular filtration rate; PSM: propensity score match; SMD: standardized mean difference.

**Table 2:** Cardiovascular adverse outcomes in the denosumab and alendronate groups.

	Denosumab		Alendronate		*p-*Value
**Overall**	***n* = 2523**		***n* = 2523**		
**Any CVD, n (%)**	**201**	**(7.97)**	**170**	**(6.74)**	**0.0945**
Myocardial infarction	26	(1.03)	25	(0.99)	0.8881
Ischemic stroke	131	(5.19)	113	(4.48)	0.2375
Congestive heart failure	75	(2.97)	55	(2.18)	0.0755
**Time to event, years**					
Mean ± SD	1.57	±1.23	1.58	±1.21	
Median (25th, 75th)	1.31	(0.54, 2.40)	1.31	(0.65, 1.31)	
**MPR of treatment ≥60%**	***n* = 1608**		***n* = 660**		
**Any CVD, n (%)**	**146**	**(9.08)**	**68**	**(10.30)**	**0.3653**
Myocardial infarction	16	(1.00)	11	(1.67)	0.1804
Ischemic stroke	94	(5.85)	46	(6.97)	0.3124
Congestive heart failure	59	(3.67)	23	(3.48)	0.8309
**Time to event, years**					
Mean ± SD	1.29	±1.14	1.01	±1.01	
Median (25th, 75th)	0.99	(0.38, 1.77)	0.77	(0.20, 1.49)	
**MPR of medications <60%**	***n* = 915**		***n* = 1863**		
**Any CVD, n (%)**	**55**	**(6.01)**	**102**	**(5.48)**	**0.5654**
Myocardial infarction	10	(1.09)	14	(0.75)	0.3608
Ischemic stroke	37	(4.04)	67	(3.60)	0.5594
Congestive heart failure	16	(1.75)	32	(1.72)	0.9530
**Time to event, years**					
Mean ± SD	2.31	±1.14	1.96	±1.19	
Median (25th, 75th)	2.48	(1.46, 3.01)	1.90	(1.02, 2.69)	

CVD: cardiovascular disease; MPR: medication possession rate.

**Table 3:** Factors associated with risk of composite outcome of cardiovascular disease.

Variables	Overall			MPR of Medications ≥60% ^#^			MPR of Medications <60%		
	AHR	95% CI	*p-*Value	AHR	95% CI	*p-*Value	AHR	95% CI	*p-*Value
**Denosumab (vs. Alendronate)**	1.03	(0.84, 1.26)	0.8071	0.74	(0.55, 1.00)	0.0493	1.04	(0.73, 1.50)	0.8178
**Median dose of treatment**									
≥ median dose (vs. < median dose)	2.37	(1.91, 2.93)	<0.0001	2.59	(1.57, 4.26)	0.0002	1.65	(1.11, 2.45)	0.0130
**Age group**									
≥65 (vs. <65) years	2.84	(1.93, 4.16)	<0.0001	2.92	(1.76, 4.86)	<0.0001	2.75	(1.54, 4.92)	0.0006
**Sex**									
Male (vs. Female)	1.31	(1.00, 1.71)	0.0479	1.15	(0.81, 1.64)	0.4281	1.40	(0.94, 2.10)	0.0984
**Baseline eGFR group**									
<60 (vs. ≥60) mL/min/1.73 m^2^	1.77	(1.43, 2.20)	<0.0001	1.74	(1.31, 2.32)	0.0001	1.83	(1.32, 2.53)	0.0003
**CVD-related risks** *									
1–2 (vs. 0)	1.92	(1.42, 2.58)	<0.0001	1.81	(1.23, 2.64)	0.0024	2.12	(1.31, 3.44)	0.0023
≥3 (vs. 0)	1.99	(1.38, 2.86)	0.0002	2.09	(1.29, 3.39)	0.0027	1.94	(1.09, 3.46)	0.0249
**Baseline comorbidities**									
Thyroid function abnormal	0.85	(0.38, 1.93)	0.7038	0.28	(0.04, 2.03)	0.2087	1.43	(0.58, 3.53)	0.4447
Obstructive sleep apnea	2.12	(0.93, 4.83)	0.0737	1.54	(0.56, 4.20)	0.4015	4.20	(1.00, 17.71)	0.0503
Fracture	1.32	(1.07, 1.62)	0.0100	1.26	(0.96, 1.67)	0.0985	1.44	(1.05, 1.98)	0.0245
**Concomitant medications**									
Anti-thrombotic agents	1.69	(1.34, 2.12)	<0.0001	1.46	(1.07, 1.99)	0.0172	2.04	(1.45, 2.87)	<0.0001
Lipid-lowering agents	0.63	(0.49, 0.81)	0.0003	0.55	(0.39, 0.77)	0.0006	0.76	(0.52, 1.11)	0.1521
Anti-diabetic agents	1.37	(1.05, 1.79)	0.0191	1.16	(0.80, 1.68)	0.4337	1.63	(1.10, 2.40)	0.0142
Anti-hypertensive agents	0.74	(0.58, 0.95)	0.0164	0.64	(0.46, 0.89)	0.0072	0.87	(0.59, 1.27)	0.4623
Other osteoporosis therapy	0.60	(0.37, 0.97)	0.0366	0.44	(0.16, 1.18)	0.1019	0.68	(0.39, 1.19)	0.1803

AHR: adjusted hazard ratio; CVD: cardiovascular disease; eGFR: estimated glomerular filtration rate; MPR: medication possession rate. * CVD-related risks: sum of baseline comorbid conditions (diabetes, hypertension, hyperlipidemia) and use of prior medications (anti-thrombotic, lipid-lowering, antidiabetic, and antihypertensive agents). # In patients with MPR ≥60%, AHR was 0.42 (95%CI, 0.18–0.97; *p* = 0.0415) for risk of myocardial infarction development (denosumab vs. alendronate); 0.72 (95%CI, 0.5–1.04; *p* = 0.0805) for ischemic stroke; and 0.74 (95%CI, 0.44–1.24; *p* = 0.2534) for congestive heart failure.

**Table 4:** Renal outcomes in the denosumab and alendronate groups during the follow-up period.

	Denosumab (*n* = 2523)	Alendronate (*n* = 2523)	*p-*Value
**Decline in eGFR (per year),**			
Mean ± SD	-0.25 ± 30.91	1.46 ± 48.24	
Median (25th, 75th)	0.71 (-3.41,4.90)	0.38 (−3.30,4.90)	
**Decline of eGFR ≥ 30%**			
Event, n (%)	302 (11.97)	257 (10.19)	0.0436
Time to event, years, mean ± SD	2.42 ± 1.58	2.39 ± 1.58	0.7564
**Occurrence of AKI**			
Even, n (%)	96 (47.06)	51 (35.66)	0.0345
Time to event, years, mean ± SD	2.42 ± 1.59	2.28 ± 1.42	0.5215

AKI: acute kidney injury; eGFR: estimated glomerular filtration rate.

## Supplementary Materials

The following are available online at https://www.mdpi.com/2077-0383/8/7/932/s1, Figure S1: Trend of eGFR between denosumab and alendronate groups, Table S1: Operational definitions for study clinical conditions and medication uses, Table S2: Medication uses between denosumab and alendronate groups, Table S3: Baseline characteristics between denosumab and alendronate users with MPR ≥ 60%.

## References

[1] Sprini D., Rini G.B., Di Stefano L., Cianferotti L., Napoli N. (2014). Correlation between osteoporosis and cardiovascular disease. Clin. Cases Miner. Bone Metab..

[2] Laroche M., Pécourneau V., Blain H., Breuil V., Chapurlat R., Cortet B., Sutter B., Degboe Y. (2017). Osteoporosis and ischemic cardiovascular disease. Jt. Bone Spine.

[3] Lampropoulos C.E., Papaioannou I., D’cruz D.P. (2012). Osteoporosis—A risk factor for cardiovascular disease?. Nat. Rev. Rheumatol..

[4] Chen S.-J., Lin C.-S., Lin C.-L., Kao C.-H. (2015). Osteoporosis is associated with high risk for coronary heart disease: A population-based cohort study. Medicine.

[5] Jamal S., West S., Miller P. (2012). Fracture risk assessment in patients with chronic kidney disease. Osteoporos. Int..

[6] Naylor K.L., McArthur E., Leslie W.D., Fraser L.-A., Jamal S.A., Cadarette S.M., Pouget J.G., Lok C.E., Hodsman A.B., Adachi J.D. (2014). The three-year incidence of fracture in chronic kidney disease. Kidney Int..

[7] Mathew R.O., Bangalore S., Lavelle M.P., Pellikka P.A., Sidhu M.S., Boden W.E., Asif A. (2017). Diagnosis and management of atherosclerotic cardiovascular disease in chronic kidney disease: A review. Kidney Int..

[8] Liberman M., Pesaro A.E.P., Carmo L.S., Serrano C.V. (2013). Vascular calcification: Pathophysiology and clinical implications. Einstein (São Paulo).

[9] Disthabanchong S. (2012). Vascular calcification in chronic kidney disease: Pathogenesis and clinical implication. World J. Nephrol..

[10] Kidney Disease: Improving Global Outcomes (KDIGO) CKD-MBD Work Group (2009). KDIGO clinical practice guideline for the diagnosis, evaluation, prevention, and treatment of Chronic Kidney Disease-Mineral and Bone Disorder (CKD-MBD). Kidney Int. Suppl..

[11] Karwowski W., Naumnik B., Szczepański M., Myśliwiec M. (2012). The mechanism of vascular calcification–a systematic review. Med. Sci. Monit. Int. Med J. Exp. Clin. Res..

[12] Ndip A., Wilkinson F.L., Jude E.B., Boulton A.J., Alexander M.Y. (2014). RANKL–OPG and RAGE modulation in vascular calcification and diabetes: Novel targets for therapy. Diabetologia.

[13] Kearns A.E., Khosla S., Kostenuik P.J. (2007). Receptor activator of nuclear factor κB ligand and osteoprotegerin regulation of bone remodeling in health and disease. Endocr. Rev..

[14] Boyce B.F., Xing L. (2008). Functions of RANKL/RANK/OPG in bone modeling and remodeling. Arch. Biochem. Biophys..

[15] Nie B., Zhou S.-Q., Fang X., Zhang S.-Y., Guan S.-M. (2015). The function and meaning of receptor activator of NF-κB ligand in arterial calcification. J. Huazhong Univ. Sci. Technol..

[16] Kiechl S., Schett G., Schwaiger J., Seppi K., Eder P., Egger G., Santer P., Mayr A., Xu Q., Willeit J. (2007). Soluble receptor activator of nuclear factor-kB ligand and risk for cardiovascular disease. Circulation.

[17] Helas S., Goettsch C., Schoppet M., Zeitz U., Hempel U., Morawietz H., Kostenuik P.J., Erben R.G., Hofbauer L.C. (2009). Inhibition of receptor activator of NF-κB ligand by denosumab attenuates vascular calcium deposition in mice. Am. J. Pathol..

[18] Cummings S.R., Martin J.S., McClung M.R., Siris E.S., Eastell R., Reid I.R., Delmas P., Zoog H.B., Austin M., Wang A. (2009). Denosumab for prevention of fractures in postmenopausal women with osteoporosis. N. Engl. J. Med..

[19] Brown J.P., Prince R.L., Deal C., Recker R.R., Kiel D.P., De Gregorio L.H., Hadji P., Hofbauer L.C., Álvaro-Gracia J.M., Wang H. (2009). Comparison of the effect of denosumab and alendronate on BMD and biochemical markers of bone turnover in postmenopausal women with low bone mass: A randomized, blinded, phase 3 trial. J. Bone Miner. Res..

[20] Freemantle N., Satram-Hoang S., Tang E.-T., Kaur P., Macarios D., Siddhanti S., Borenstein J., Kendler D. (2012). Final results of the DAPS (Denosumab Adherence Preference Satisfaction) study: A 24-month, randomized, crossover comparison with alendronate in postmenopausal women. Osteoporos. Int..

[21] Enjuanes A., Ruiz-Gaspà S., Peris P., Ozalla D., Álvarez L., Combalia A., de Osaba M.J.M., Monegal A., Pares A., Guañabens N. (2010). The effect of the alendronate on OPG/RANKL system in differentiated primary human osteoblasts. Endocrine.

[22] Hanley D., Adachi J., Bell A., Brown V. (2012). Denosumab: Mechanism of action and clinical outcomes. Int. J. Clin. Pract..

[23] Chang Gung Memorial Hospital (2015). About CGMH: Medical Service Overview: Chang Gung Medical Foundation. http://www.webcitation.org/76ciBI4kH.

[24] Cheng T.-M. (2003). Taiwan’s new national health insurance program: Genesis and experience so far. Health Aff..

[25] Levey A.S., Bosch J.P., Lewis J.B., Greene T., Rogers N., Roth D. (1999). A more accurate method to estimate glomerular filtration rate from serum creatinine: A new prediction equation. Ann. Intern. Med..

[26] Kellum J.A., Lameire N., Aspelin P., Barsoum R.S., Burdmann E.A., Goldstein S.L., Herzog C.A., Joannidis M., Kribben A., Levey A.S. (2012). Kidney disease: Improving global outcomes (KDIGO) acute kidney injury work group. KDIGO clinical practice guideline for acute kidney injury. Kidney Int. Suppl..

[27] Parsons L.S. Performing a 1: N Case-Control Match on Propensity Score. Proceedings of the 29th Annual SAS Users Group International Conference.

[28] Austin P.C. (2009). Using the standardized difference to compare the prevalence of a binary variable between two groups in observational research. Commun. Stat. Simul. Comput..

[29] Hofbauer L., Brueck C., Shanahan C., Schoppet M., Dobnig H. (2007). Vascular calcification and osteoporosis—From clinical observation towards molecular understanding. Osteoporos. Int..

[30] Kiechl S., Schett G., Wenning G., Redlich K., Oberhollenzer M., Mayr A., Santer P., Smolen J., Poewe W., Willeit J. (2004). Osteoprotegerin is a risk factor for progressive atherosclerosis and cardiovascular disease. Circulation.

[31] Tschiderer L., Willeit J., Schett G., Kiechl S., Willeit P. (2017). Osteoprotegerin concentration and risk of cardiovascular outcomes in nine general population studies: Literature-based meta-analysis involving 26,442 participants. PLoS ONE.

[32] Sandberg W.J., Yndestad A., Øie E., Smith C., Ueland T., Ovchinnikova O., Robertson A.-K.L., Müller F., Semb A.G., Scholz H. (2006). Enhanced T-cell expression of RANK ligand in acute coronary syndrome: Possible role in plaque destabilization. Arterioscler. Thromb. Vasc. Biol..

[33] Collin-Osdoby P. (2004). Regulation of vascular calcification by osteoclast regulatory factors RANKL and osteoprotegerin. Circ. Res..

[34] Crisafulli A., Micari A., Altavilla D., Saporito F., Sardella A., Passaniti M., Raffa S., D’anneo G., Lucà F., Mioni C. (2005). Serum levels of osteoprotegerin and RANKL in patients with ST elevation acute myocardial infarction. Clin. Sci..

[35] Samelson E.J., Miller P.D., Christiansen C., Daizadeh N.S., Grazette L., Anthony M.S., Egbuna O., Wang A., Siddhanti S.R., Cheung A.M. (2014). RANKL inhibition with denosumab does not influence 3-year progression of aortic calcification or incidence of adverse cardiovascular events in postmenopausal women with osteoporosis and high cardiovascular risk. J. Bone Miner. Res..

[36] Kendler D., McClung M., Freemantle N., Lillestol M., Moffett A., Borenstein J., Satram-Hoang S., Yang Y.-C., Kaur P., Macarios D. (2011). Adherence, preference, and satisfaction of postmenopausal women taking denosumab or alendronate. Osteoporos. Int..

[37] Mascelli M.A., Zhou H., Sweet R., Getsy J., Davis H.M., Graham M., Abernethy D. (2007). Molecular, biologic, and pharmacokinetic properties of monoclonal antibodies: Impact of these parameters on early clinical development. J. Clin. Pharmacol..

[38] Lewiecki E.M. (2008). Denosumab: An investigational drug for the management of postmenopausal osteoporosis. Biol. Targets Ther..

[39] Jamal S.A., Ljunggren Ö., Stehman-Breen C., Cummings S.R., McClung M.R., Goemaere S., Ebeling P.R., Franek E., Yang Y.-C., Egbuna O.I. (2011). Effects of denosumab on fracture and bone mineral density by level of kidney function. J. Bone Miner. Res..

[40] Sadowski C.A., Spencer T., Yuksel N. (2011). Use of oral bisphosphonates by older adults with fractures and impaired renal function. Can. J. Hosp. Pharm..

[41] Thongprayoon C., Acharya P., Aeddula N.R., Torres-Ortiz A., Bathini T., Sharma K., Ungprasert P., Watthanasuntorn K., Suarez M.L.G., Salim S.A. (2019). Effects of denosumab on bone metabolism and bone mineral density in kidney transplant patients: A systematic review and meta-analysis. Arch. Osteoporos..

[42] Wilson L.M., Rebholz C.M., Jirru E., Liu M.C., Zhang A., Gayleard J., Chu Y., Robinson K.A. (2017). Benefits and Harms of Osteoporosis Medications in Patients With Chronic Kidney Disease: A Systematic Review and Meta-analysis. Ann. Intern. Med..

[43] Yamasaki M., Yuasa T., Uehara S., Fujii Y., Yamamoto S., Masuda H., Fukui I., Yonese J. (2016). Improvement of renal function by changing the bone-modifying agent from zoledronic acid to denosumab. Int. J. Clin. Oncol..

[44] Kurata T., Nakagawa K. (2012). Efficacy and safety of denosumab for the treatment of bone metastases in patients with advanced cancer. Jpn. J. Clin. Oncol..

[45] Raje N., Terpos E., Willenbacher W., Shimizu K., García-Sanz R., Durie B., Legieć W., Krejčí M., Laribi K., Zhu L. (2018). Denosumab versus zoledronic acid in bone disease treatment of newly diagnosed multiple myeloma: An international, double-blind, double-dummy, randomised, controlled, phase 3 study. Lancet Oncol..

[46] Banerjee D., Asif A., Striker L., Preston R.A., Bourgoignie J.J., Roth D. (2003). Short-term, high-dose pamidronate-induced acute tubular necrosis: The postulated mechanisms of bisphosphonate nephrotoxicity. Am. J. Kidney Dis..

[47] Markowitz G.S., Fine P.L., Stack J.I., Kunis C.L., Radhakrishnan J., Palecki W., Park J., Nasr S.H., Hoh S., Siegel D.S. (2003). Toxic acute tubular necrosis following treatment with zoledronate (Zometa). Kidney Int..

[48] Miller P.D., Jamal S.A., Evenepoel P., Eastell R., Boonen S. (2013). Renal safety in patients treated with bisphosphonates for osteoporosis: A review. J. Bone Miner. Res..

