# Supplementary material for: Comparison of the Effects of Denosumab and Alendronate on Cardiovascular and Renal Outcomes in Osteoporotic Patients
Source: J Clin Med. 2019 Jun 28;8(7):932. doi: 10.3390/jcm8070932 (PMC6678904; doi:10.3390/jcm8070932)
Supplement: Supplementary file 1 [file jcm-08-00932-s001.pdf]

Table S1. Operational definitions for study clinical conditions and medication uses

Disease condition	ICD-9/ ICD-10 and the definition
**Baseline history (≥ two times in outpatient or one inpatient diagnosis within 1 year prior to the index date)**	
End stage renal disease requiring dialysis	**Hemodialysis**<br>ICD-9-CM PCS: 39.95<br>ICD-10-CM: 5A1D60Z, 5A1D00Z<br>Billing code: 58001C, 58002CB, 58014C, 58018C, 58019C, 58020C, 58021C, 58022C, 58023C, 58024C, 58025C, 58027C, 58029C, 58030B<br>**Peritoneal dialysis**<br>ICD-9-CM/PCS: 54.98<br>ICD-10-CM: 3E1M39Z<br>Billing code: 58002C, 58009B, 58010A, 58010B, 58011A, 58011AB, 58017B, 58017C, 58011B, 58011C, 58012A, 58012B, 58028C
Kidney transplantation	ICD-9-CM: V42.0<br>ICD-10-CM: Z94
Myocardial infarction and ischemic heart disease[1]	ICD-9-CM: 410-414<br>ICD-10-CM: I21, I22, I24, I25
Coronary artery bypass graft (CABG) [2]	ICD-9 PCS: 36.1<br>ICD-10 CM: Z95.1<br>ICD-10 PCS: 021
Percutaneous coronary intervention (PCI) [3]	ICD-9 PCS: 00.66, 36.01,36.02, 36.05, 36.06, 36.07, 17.55<br>ICD-10 PCS: 0270, 0271, 0272, 0273, 3E07
Stroke[4]	ICD-9-CM: 433-437<br>ICD-10-CM: I63, I65,I66, I67.0~I67.2, I67.4~I67.82, I67.841~I67.9, I68, G45.0~G45.2, G45.4~G45.9, G46
Congestive heart failure [4]	ICD-9-CM: 428, 428.x<br>ICD-10-CM: I5020-I5023, I5030-I5033, I5040-I5043, I509, I501
Cancer	ICD-9-CM: 140-208<br>ICD-10-CM: Cxxx, z5112
**Outcome (≥ 1 hospital discharge diagnosis during the study period)**	
Acute myocardial infarction [5]	ICD-9-CM: 410.xx, 411.89<br>ICD-10-CM: R0989, I21, I22, I248, I249
Ischemic stroke [6]	ICD-9-CM: 433, 434, 435.x, 436, 437, 437.0, 437.0A, 437.1, 437.2, 437.8, 437.9, 438.xx<br>ICD-10-CM: I63, I65, I66, I67.0~I67.2, I67.4~I67.82, I67.841~I67.9, I68, G45.0~G45.2, G45.4~G45.9, G46, I69
Congestive heart failure[4]	ICD-9-CM: 428, 428.x<br>ICD-10-CM: I50

Acute kidney injury [7]	Increase in serum creatinine (SCr) by ≥0.3 mg/dl within 48 hours, or increase in SCr to ≥ 1.5 times baseline within 7 days, or<br>increase in serum creatinine to ≥ 4.0 mg/dl
**Laboratory test**	
eGFR value	Measured per 6 months, mean value is averaged by measurement in the last 3 months<br>If inavailable, mean value is averaged by measurement in the prior 3 months
Changes in eGFR per year	baseline eGFR minus the last eGFR then divided by years of follow-up<br>baseline eGFR: mean value 6 months before index day<br>last eGFR: mean value 6 months after index day
**Medications**	**Anatomical Therapeutic Chemical (ATC) code**
Anti-thrombotic agents	B01A
Lipid-lowering agents	C10AA, C10AB, C10AC, C10AX, C10BA, C10BX
Anti-diabetic agents	A10
Anti-hypertension	C09AA, C09BB, C09CA, C09DA, C09DB, C09DX, C09XA, C03AA, C03DA, C03CB01, C03CA02
Other osteoporosis therapy	
1. Other bisphosphonates (except alendronate)	M05BA02, M05BA03, M05BA06, M05BA07, M05BA08
2. Raloxifene	G03XC01
3. Teriparatide	H05AA02
4. Calcitonin preparations	H05BA01, H05BA02, H05BA03
**Prior use of medication was identified ≤365 days before the index date with ≥28 days of supply**<br>**Concomitant use of medication for ≥28 days of supply was identified between the index date and the earliest date of event of interest.**	

eGFR: estimated glomerular filtration rate; ICD-9-CM/PCS: International Classification of Diseases, Ninth Revision, Clinical Modification and Procedure Classification System; ICD-10-CM/PCS: International Classification of Diseases, Tenth Revision, Clinical Modification and Procedure Classification System.

References

1. Metcalfe, A.; Neudam, A.; Forde, S.; Liu, M.; Drosler, S.; Quan, H.; Jetté, N. Case definitions for acute myocardial infarction in administrative databases and their impact on in-hospital mortality rates. *Health services research* **2013**, *48*, 290-318.
2. Davis, L.A.; Mann, A.; Cannon, G.W.; Mikuls, T.R.; Reimold, A.M.; Caplan, L. Validation of diagnostic and procedural codes for identification of acute cardiovascular events in US veterans with rheumatoid arthritis. *EGEMS* **2013**, *1*.
3. Ben-Josef, G.; Ott, L.S.; Spivack, S.B.; Wang, C.; Ross, J.S.; Shah, S.J.; Curtis, J.P.; Kim, N.; Krumholz, H.M.; Bernheim, S.M. Payments for acute myocardial infarction episodes-of-care initiated at hospitals with and without interventional capabilities. *Circulation: Cardiovascular*

Quality and Outcomes **2014**, *7*, 882-888.
4. Hartle, J.E.; Tang, X.; Kirchner, H.L.; Bucaloiu, I.D.; Sartorius, J.A.; Pogrebnaya, Z.V.; Akers, G.A.; Carnero, G.E.; Perkins, R.M. Bisphosphonate therapy, death, and cardiovascular events among female patients with CKD: a retrospective cohort study. *American Journal of Kidney Diseases* **2012**, *59*, 636-644.
5. Cheng, C.-L.; Lee, C.-H.; Chen, P.-S.; Li, Y.-H.; Lin, S.-J.; Yang, Y.-H.K. Validation of acute myocardial infarction cases in the national health insurance research database in Taiwan. *Journal of epidemiology* **2014**, *24*, 500-507.
6. Hsieh, C.-Y.; Chen, C.-H.; Li, C.-Y.; Lai, M.-L. Validating the diagnosis of acute ischemic stroke in a National Health Insurance claims database. *Journal of the Formosan Medical Association* **2015**, *114*, 254-259.
7. Kellum, J.A.; Lameire, N.; Aspelin, P.; Barsoum, R.S.; Burdmann, E.A.; Goldstein, S.L.; Herzog, C.A.; Joannidis, M.; Kribben, A.; Levey, A.S. Kidney disease: improving global outcomes (KDIGO) acute kidney injury work group. KDIGO clinical practice guideline for acute kidney injury. *Kidney international supplements* **2012**, *2*, 1-138.

Table S2. Medication uses between denosumab and alendronate groups

	Overall n	Denosumab (n=2523) n (%)	Alendronate (n=2523) n (%)	*p-value*
**Treatment group switching**				<.0001
Yes	1798	73 (2.06)	1725 (13.39)	
No	14621	3463 (97.94)	11158 (86.61)	
**Concomitant medications**				
**Anti-thrombotic agents**	1122	574 (22.75)	548 (21.72)	0.3787
Oral anticoagulants	170	83 (3.29)	87 (3.45)	0.7550
Anti-platelets	987	509 (20.17)	478 (18.95)	0.2712
Aspirin	733	376 (14.90)	357 (14.15)	0.4478
Heparin	6	4 (0.16)	2 (0.08)	0.4139
**Lipid-lowering agents**	1483	760 (30.12)	723 (28.66)	0.2529
Statins	1403	715 (28.34)	688 (27.27)	0.3962
Fibrates	144	73 (2.89)	71 (2.81)	0.8657
Others	46	28 (1.11)	18 (0.71)	0.1386
**Anti-diabetic agents**	1124	548 (21.72)	576 (22.83)	0.3435
**Anti-hypertension**	1886	955 (37.85)	931 (36.90)	0.4850
ACEI / ARBs / Aliskiren	1776	900 (35.67)	876 (34.72)	0.4793
Diuretics	296	150 (5.95)	146 (5.79)	0.8106
**Other osteoporosis therapy**	381	134 (5.31)	247 (9.79)	<.0001
Bisphosphonates	21	5 (0.20)	16 (0.63)	0.0162
Ibandronate	3	1 (0.04)	2 (0.08)	0.5636
Risedronate	16	3 (0.12)	13 (0.52)	0.0123
Raloxifene	228	71 (2.81)	157 (6.22)	<.0001
Teriparatide	157	71 (2.81)	86 (3.41)	0.2239
Calcitonin preparations	8	0 (0.00)	8 (0.32)	0.0046

Concomitant medication use for ≥ 28 days of supply was categorized from the index date to the primary outcome of composite cardiovascular event or censored date.

ACEI : angiotensin converting enzyme inhibitors; ARBs : angiotensin receptor blockers; MPR: medication possession rate.

Table S3. Baseline characteristics between denosumab and alendronate users with MPR ≥ 60%

	Overall	Denosumab (n=1608)	Alendronate (n=660)	
	n	n (%)	n (%)	*p-value*
**Age, mean (SD) years,**	2268	71.85 (10.00)	72.16 (10.23)	0.5020
**Sex**				0.6782
Male	404	283 (17.60)	121 (18.33)	
Female	1864	1325 (82.40)	539 (81.67)	
**eGFR group**				0.5222
<60	584	408 (25.37)	176 (26.67)	
≥60	1684	1200 (74.63)	484 (73.33)	
**Baseline comorbidities**				
Acute myocardial infarction	0	0 (0.00)	0 (0.00)	-
Congestive heart failure	0	0 (0.00)	0 (0.00)	-
Peripheral vascular diseases	42	30 (1.87)	12 (1.82)	0.9393
Cerebral vascular accident				
Dementia	109	73 (4.54)	36 (5.45)	0.3549
Pulmonary disease	276	185 (11.50)	91 (13.79)	0.1309
Connective tissue disorder	129	79 (4.91)	50 (7.58)	0.0129
Peptic ulcer	430	304 (18.91)	126 (19.09)	0.9185
Liver diseases	332	220 (13.68)	112 (16.97)	0.0442
Diabetes	548	374 (23.26)	174 (26.36)	0.1166
Diabetes complications	178	121 (7.52)	57 (8.64)	0.3713
Paraplegia	18	13 (0.81)	5 (0.76)	0.9013
Renal disease	217	138 (8.58)	79 (11.97)	0.0127
Cancer	0	0 (0.00)	0 (0.00)	-
Severe liver diseases	13	7 (0.44)	6 (0.91)	0.1746
Metastatic cancer	1	1 (0.06)	0 (0.00)	0.5217
HIV	0	0 (0.00)	0 (0.00)	-
Hypertension	1092	771 (47.95)	321 (48.64)	0.7656
Hyperlipidemia	636	447 (27.80)	189 (28.64)	0.6866
Thyroid function abnormal	44	33 (2.05)	11 (1.67)	0.5454
Obstructive sleep apnea	68	45 (2.80)	23 (3.48)	0.3840
Fracture	775	559 (34.76)	216 (32.73)	0.3530
**Prior medications (≦365 days before the index date, ≧28 days use)**				

**Anti-thrombotic agents**				
Oral anticoagulants	52	42 (2.61)	10 (1.52)	0.1129
Anti-platelet agents	374	255 (15.86)	119 (18.03)	0.2055
Aspirin	289	199 (12.38)	90 (13.64)	0.4134
**Lipid-lowering agents**				
Statins	511	350 (21.77)	161 (24.39)	0.1736
Fibrates	48	37 (2.30)	11 (1.67)	0.3404
Others	13	9 (0.56)	4 (0.61)	0.8943
**Anti-diabetics**	466	316 (19.65)	150 (22.73)	0.0996
**Anti-hypertensive**				
ACEI / ARBs / Aliskiren	709	494 (30.72)	215 (32.58)	0.3869
Diuretics	104	70 (4.35)	34 (5.15)	0.4091
**Osteoporosis therapy**				
Bisphosphonates	1	1 (0.06)	0 (0.00)	-
Raloxifene (SERM)	84	67 (4.17)	17 (2.58)	0.0684
Forteo (Teriparatide)	40	29 (1.80)	11 (1.67)	0.8221
Calcitonin preparations	22	15 (0.93)	7 (1.06)	0.7779

ACEI: angiotensin converting enzyme inhibitors; ARBs: angiotensin receptor blockers; MPR: medication possession rate.

Figure S1. Trend of eGFR between denosumab and alendronate groups

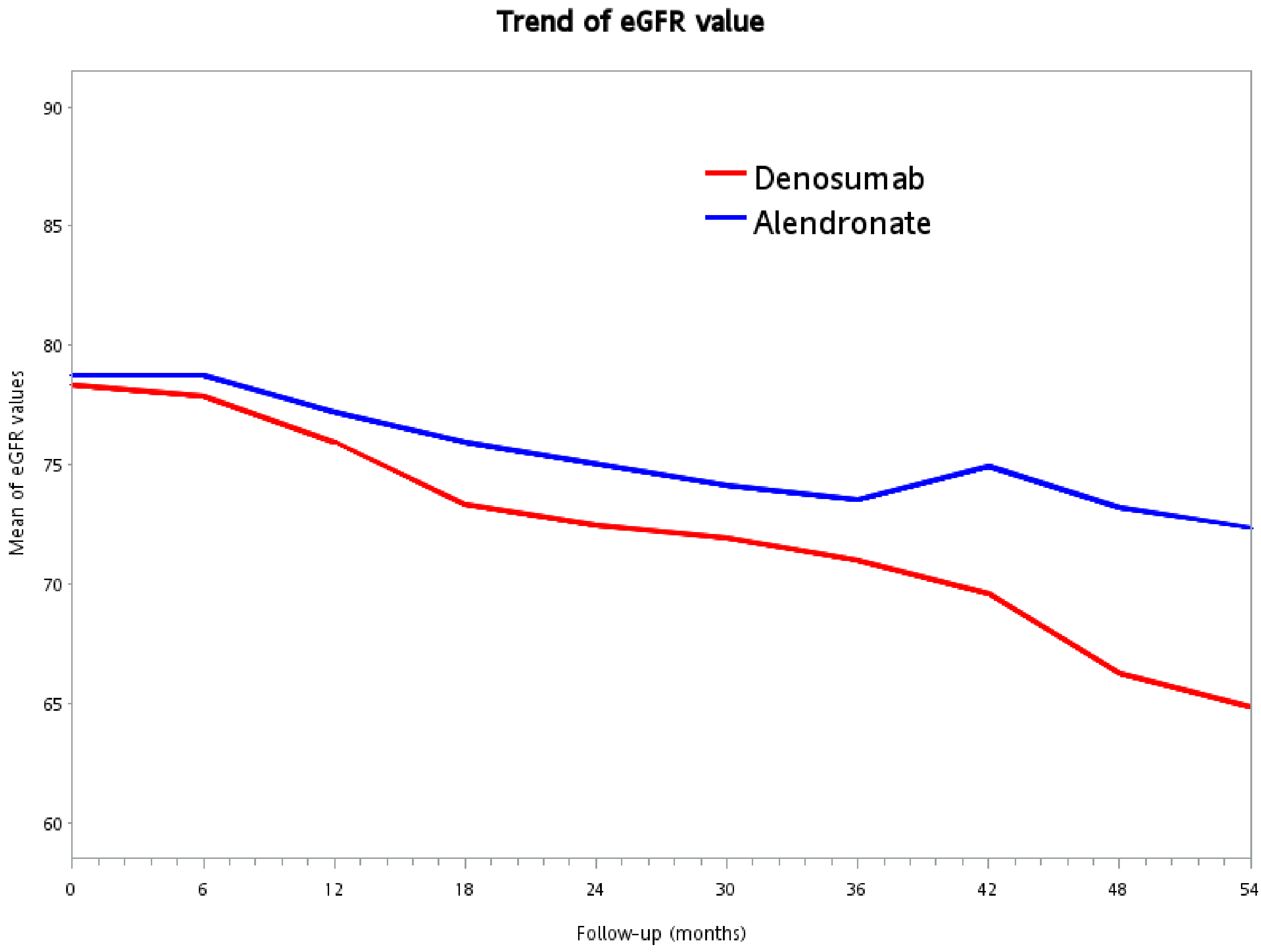

eGFR: estimated glomerular filtration rate
